# Understanding interdisciplinary perspectives of plant intelligence: Is it a matter of science, language, or subjectivity?

**DOI:** 10.1186/s13002-022-00539-3

**Published:** 2022-05-30

**Authors:** Jennifer Khattar, Paco Calvo, Ina Vandebroek, Camilla Pandolfi, Farid Dahdouh-Guebas

**Affiliations:** 1 Systems Ecology and Resource Management, Department of Organism Biology, Faculté des Sciences, Université Libre de Bruxelles - ULB, Avenue F.D. Roosevelt 50, CPi 264/1, 1050 Brussels, Belgium; 2 Ecology and Biodiversity, Laboratory of Plant Biology and Nature Management, Biology Department, Vrije Universiteit Brussel - VUB, Pleinlaan 2, VUB-APNA-WE, 1050 Brussels, Belgium; 3 International Laboratory of Plant Neurobiology (LINV), Department of Plant, Soil and Environmental Science, Università degli Studi di Firenze, Viale delle Idee 30, 50019 Sesto Fiorentino, Tuscany Italy; 4 Minimal Intelligence Lab, Department of Philosophy, University of Murcia, 30100 Murcia, Spain; 5 Faculty of Science and Technology, Department of Life Sciences and Natural Products Institute, The University of the West Indies, Mona Campus, Kingston, Jamaica; 6 Institute of Economic Botany, The New York Botanical Garden, 2900 Southern Boulevard, Bronx, NY 10458 USA; 7 Interfaculty Institute of Social-Ecological Transitions - iiTSE, Université Libre de Bruxelles - ULB, Brussels, Belgium

**Keywords:** Plant intelligence, Traditional knowledge (TK), Plant sciences, Western scientific knowledge, Interdisciplinarity

## Abstract

**Background:**

Evidence suggests that plants can behave intelligently by exhibiting the ability to learn, make associations between environmental cues, engage in complex decisions about resource acquisition, memorize, and adapt in flexible ways. However, plant intelligence is a disputed concept in the scientific community. Reasons for lack of consensus can be traced back to the history of Western philosophy, interpretation of terminology, and due to plants lacking neurons and a central nervous system. Plant intelligence thus constitutes a novel paradigm in the plant sciences. Therefore, the perspectives of scientists in plant-related disciplines need to be investigated in order to gain insight into the current state and future development of this concept.

**Methods:**

This study analyzed opinions of plant intelligence held by scientists from different plant-related disciplines, including ethnobiology and other biological sciences, through an online questionnaire.

**Results:**

Our findings show that respondents’ personal belief systems and the frequency of taking into account other types of knowledge, such as traditional knowledge, in their own field(s) of study, were associated with their opinions of plant intelligence. Meanwhile, respondents’ professional expertise, background (discipline), or familiarity with evidence provided on plant intelligence did not affect their opinions.

**Conclusions:**

This study emphasizes the influential role of scientists’ own subjective beliefs. In response, two approaches could facilitate transdisciplinary understanding among scientists: (1) effective communication designed to foster change in agreement based on presented information; and (2) holding space for an interdisciplinary dialogue where scientists can express their own subjectivities and open new opportunities for collaboration.

## Introduction

Paradigm shifts in worldviews and perceptions are a part of human cultural evolution [1, 2]. Human–nature relationships, whether agronomic, leisurely, experimental, or spiritual, are a reflection of people’s perception of nature (including plants), contained within their cultural and environmental domains [3–5]. Plant scientists and philosophers have proposed a new paradigm in the way plants are perceived by demonstrating the potential of plants to have agency, individuality, and even intelligence [6–8].

### What is plant intelligence?

A relatively newly termed concept, plant intelligence, refers to “*any type of intentional and flexible behavior that is beneficial and enables the organism to achieve its goal*” [9]. Plant behavior can be defined as “*a response to an event or environmental change during the course of an individual’s lifetime*” [10]. While animals use direct movement as a behavioral response, plant behavior is characterized by changes in growth and development. Phenotypic traits in response to environmental changes can either be hardwired (inflexible responses that are genetically encoded) or soft-wired (flexible responses that are flexible and dependent on changing environments). Beneficial behaviors that are adaptive (i.e., flexible) are the soft-wired, phenotypically plastic responses, capable of integrating multiple information signals and channeling an optimal global response [11]. Notably, a simple one-to-one response to an external stimulus is not considered intelligent behavior. The author in [10] also gives a list of examples to differentiate between inflexible and flexible behaviors.

### Examples of plant intelligence

Possible examples of intelligence in plants include key features that involve the ability to problem-solve in a flexible manner, anticipate the future, store memory, learn, communicate, and ultimately be goal-oriented [11–13]. Various plant behaviors can indicate intelligence (e.g., [8, 9, 14–16]). For example, induced volatile defenses against herbivores have to be produced at the appropriate time, concentration, and amount, demonstrating a plant’s flexibility (i.e., adaptability). These behavioral responses in turn affect, and are affected, by neighboring plants, shaping plant communities [17, 18]. Another example of flexible plant behavior is the capability to manipulate the concentration of secondary metabolites and control the quality of defense provided by species-specific ants [19]. An example of the ability to anticipate the future is how plants rely on light cues to remember the exact number of warm days or daylight hours (i.e., photoperiodic control) that have passed to develop their leaves and flower [20, 21]. Moreover, carnivorous plants such as the Venus fly trap, *Dionaea muscipula* J.Ellis, are able to “count” and “memorize” the number of mechanical stimulations to ensure trapping success [14]. This plant species and others need to store and retrieve this information when required. There exist examples of many other ways in which plants can be phenotypically flexible to prepare for the future [22]. Furthermore, it seems that plants can also learn to make new associations through multiple cues and respond adaptively [16]. Various types of foraging behavior for sunlight and nutrients exemplify how plants can be goal-oriented [23–26]. These types of key features of intelligence are independent of taxa and of a central nervous system. These behaviors ultimately have a physiological basis which involves electrochemistry [27, 28]. To assess a plant’s intelligence is to observe the behavior from a global perspective, i.e., what the behavior represents, and ask whether it represents any of the key features of intelligence (the capacity to adapt in a flexible manner). The mechanisms and physiological basis are essential to answer “How?”.

### Current views on plant intelligence

Plant intelligence contrasts the conventional view of plants, in which they behave passively without any intention in their responses. Because the developing framework of plant intelligence is conceptually new in the plant sciences, several authors have shared their disagreements in the literature [29–32]. To name a few, these authors argue that plants do not have nerves and do not make conscious decisions, characterizing plants as intelligent is unnecessary, and that the “individuality” of a plant is difficult to identify. To briefly address the latter argument, what is considered a plant individual remains an issue of debate. According to some authors (e.g., [8]), a plant individual is at the level of the genet (i.e., coming from the same genetic source). Plant intelligence is a trait characterizing the whole plant body in pursuit of maximizing its fitness as well as multiple individuals behaving as a collective group [13, 33]. In addition, individuality can also be observed by a plant’s ability to recognize self from non-self as well as kin [34]. Scientists and philosophers who advocate for plant intelligence argue that the main reasons why the idea is controversial are due to historical perceptions about plants and their hierarchical place in relation to other organisms that still predominate in society [7], non-consensus in terminological definitions, and traditional views of the cognitive sciences [35]. Traditional cognitive theories of intelligence are built on foundations that exclude plants because they are implicitly or explicitly zoocentric [36, 37] and neurocentric [38]. The literature in support of plant intelligence advocates for the autonomy of plants in their ability to make complex decisions in an ecological and purposeful way to improve their chance of survival [6]. For clarification, these literature sources do not refer to “intelligence” as intelligent design.

### Nature perceived by different forms of knowing and knowledge

Western scientific knowledge is based on rational, materialistic, reductionist, and objective methods for understanding the world [39]. One of the primary ways in which natural phenomena are observed and interpreted is based on our scientific understanding of them. However, from a non-Western cultural perspective, the ontology and epistemology of understanding natural phenomena is perceived differently, which in turn contributes to the constitution of a worldview. For example, the ontology that plants (and nature) are agents (or “*persons*” and not “*objects*”) is commonly shared in some traditional cultures and has been elaborately described in the anthropological literature [40–42]. Traditional knowledge (TK) (also known as traditional ecological knowledge or TEK) is more holistic, subjective, and qualitative than Western scientific knowledge [43]. The author in [44] has summarized TK as “*a cumulative body of knowledge, practice and belief, evolving by adaptive processes and handed down through generations by cultural transmission, about the relationship of living beings (including humans) with one another and with their environment*.” The author referred to “*traditional*” as a cultural continuity derived from historical experience [44]. Several traditional cultures believe in the autonomy of nature, treat nature as close kin and perceive nature as being alive and part of a reciprocal co-creating relationship [40–42, 45–48]. Belief in the interconnectedness of all things, such as with nature, is also shared among people who care about their spirituality [49, 50]. According to different definitions of spirituality across decades of research, authors in [51] summarized spirituality as an “*aspect of human functioning, experience, and existence which concerns the transcendent*.” Understanding natural phenomena through a spiritual lens is centrally an experiential quality that is related to one’s well-being, not explicit to being religious, and differs across cultures [51]. Whether from a scientific or traditional lens, they share a common goal of seeking to gain a more holistic perspective of the world.

We hypothesized that academic researchers in plant-related biological sciences who are more open to different forms of knowledge and ontologies, such as traditional knowledge systems (TKS) would likely be more acceptive of the idea of plant intelligence than those who are less open to TKS. We also speculated that the former would be more mindful of, and more inclined toward, applying sociocultural perspectives in their field(s) of study (i.e., ethnobiologists). As a result, we hypothesized that perspectives of plant intelligence will not only be influenced by scientific evidence and concepts of plant behavioral responses, but also pertain to scholars’ personal worldviews of nature, especially in relation to plants.

### Study objectives

Because of the increasing interest in plant intelligence and its features*,* our study was conceived as a preliminary investigation into perceptions of plant intelligence from plant scientists in the social and biological sciences. We gathered a wide range of data with the aim of bringing together multiple and contrasting perspectives to explore major themes and associations that represent scientists’ opinions of plant intelligence. Four research questions guided this study:

1. Which factors influence opinions of plant intelligent behavior?
2. Do other forms of knowledge systems, such as TKS, influence opinions of plant intelligence?
3. Can plant scientists distinguish behaviors that are indicative of plant intelligence?
4. Is the idea of animal intelligence easier to accept than that of plant intelligence?

## Methods

### Questionnaire development and administration online

A semi-structured questionnaire was developed and disseminated through an online survey platform, LimeSurvey, by the Université Libre de Bruxelles (ULB), from April to June 2019. Questions included multiple choice, open-ended, dichotomous, and Likert-scale formats. Likert-scale questions had an even-numbered analogue scale (ranging from 1 to 4 or 1 to 6) with only the two ends (anchors) of the scale verbally defined, making a neutral position (e.g., option 3 in a 5-point scale) impossible. An analogue scale was preferred as a reliable measurement for subjective attitudes that cannot be measured directly (e.g., the degree of plant intelligence) [52]. An even-numbered scale was preferred as a physical measurement of answers from study participants [53]. Most questions had the option “other” to allow flexibility for respondents who had a neutral response or who preferred not to make a clear choice.

### Questionnaire distribution

Plant scientists and graduate students whose professional background and work were related to plant sciences in any disciplinary field were invited as part of our effort to include as many individuals from a wide range of plant-related sciences. The term plant-related sciences here refers to any scientific study of plants that includes both biological and social subdisciplines, ranging from plant biology and forest ecology to ethnobotany. Target participants’ e-mails were found through systematic keywords in search engines, mainly Google Search and Google Scholar (see Appendix Table 5). A news post on the Web site of the International Laboratory of Plant Neurobiology (LINV) was posted online as an open invitation to plant scientists. Similarly, co-authors shared invitations with prospective participants through their personal networks. With every e-mail invitation, the LINV post link for the study was attached. Open invitations were also posted on social media platforms such as Twitter and Facebook. In total, more than 1500 individuals were directly contacted by e-mail to participate in the online questionnaire. The survey link was active from March 26 until May 21, 2019. By only counting e-mail invitations, the questionnaire had a participation rate of approximately 10% (*n* = 150). Participation success through online channels could not be estimated. Prior to distribution, the questionnaire was pretested among graduate students for feedback and further revision.

### Respondents’ general background

Out of a total of 150 respondents, 119 completed the questionnaire, of which 29 were graduate students. Their age ranged from 20 to 79 years (average age 44 ± 15 years). These study participants were residents of 31 different countries across Africa (*n* = 3), America (*n* = 62), Asia (*n* = 14), Australia (*n* = 7), and Europe (*n* = 63). Respondents’ professional fields of expertise came from a wide range of plant-related sciences, including plant ecology, plant biology, epigenetics, phylogenetics, evolutionary biology, paleobotany, invasion biology, phytochemistry, biotechnology, plant signaling and behavior, plant physiology and microbiology, plant biochemistry, horticulture, forest ecology, agronomy-related studies, silviculture, medicinal plant studies, taxonomy, botany, ethnopharmacology, and ethnobotany.

### Questionnaire content

The questionnaire consisted of four parts: (a) personal background information of participants, (b) personal beliefs and associations with different forms of knowledge, (c) perspectives on plant intelligence and traditional knowledge (TK), and (d) behavioral examples of plant intelligence and analogous behaviors in animals and plants. Before the start of the questionnaire, participants were asked to read a short essay of 302 words introducing the definition and concept of plant intelligent behavior.

Background information included questions about country of residence, age, profession, expertise and research interests, and years of professional experience. A psychometric scale, named “personal perspectives,” was created to understand an individual’s personal beliefs and associations with different forms of knowledge measured through a list of 12 statements, asking respondents to choose between the options: agree, disagree, or other. These were sourced and adapted from mixed methods literature related to (1) traditional knowledge (TK), especially pertaining to indigenous knowledge, (2) individual spirituality, and (3) other miscellaneous concepts that did not have a common theme (see Table 2). The concepts behind most statements were selected for their juxtaposition with Western scientific knowledge. Next, we explored whether agreement or disagreement with these statements had any significant relationship with an individual’s acceptance of plant intelligence. We hypothesized that those who are receptive to different forms of knowing and knowledge would be more accepting of plant intelligence. The questionnaire part that dealt with perspectives on plant intelligence included questions related to participants’ current knowledge of the concept of plant intelligence, their frequency of collaborations outside the plant sciences (i.e., collaborations with the humanities), their opinions on the usefulness of including TK in their field(s) of study, and their opinions on how much TK is valued by plant scientists. The final and central part of the questionnaire contained a list of short examples of plant behaviors obtained from the literature (Table 1). Each example provided essential information for study participants to answer. Respondents were asked to rank whether they considered the plant behavior example as intelligent or not. Respondents who did not consider a behavior intelligent were asked to explain why in an open text box.

Each example represented at least one key indicator of plant intelligence (either learning, memory, anticipatory and/or adaptive behavior). These behavioral examples can be summarized as follows (Table 1): (1) habituation and conditioned learning involving two specific plant behavioral studies that tested intelligence in plants analogous to classical animal behavioral studies; (2) seven examples of plant behavioral responses involving roots and aboveground plant parts; and (3) two examples of animal behaviors on camouflaging and foraging (Cephalopods and *Macaca arctoides*) and two corresponding analogous behaviors in plants (*Corydalis hemidicentra* Hand.-Mazz and *Syngonium* spp.*,* respectively). We aimed to compare analogous animal and plant behaviors in order to observe whether intelligence in animals was more likely to be accepted than plant intelligence. Two of the seven plant behavioral examples were actions representative of mere behaviors and not intelligence. Specifically, they were titled as “mycoheterotrophic parasitic plant” and “plant defense turning herbivores into cannibals.” The first is not an indicator of intelligent behavior because the example describes a fixed behavior incapable of long-term change, while the latter is actually an experimental trick not performed by the plant itself, but by the scientist(s). Even without a human agent, the chemical emission emitted by the plant does not involve adaptive behavior nor complex decision-making [55]. Both examples do not represent features of intelligence. These considerably tricky questions were included to observe if individuals could discern the difference between plant behaviors indicative of intelligence and those that were not. In other words, we tested if individuals would “score homogeneously,” i.e., answer all the behavioral examples in the same way.

### Classifying groups

#### Professional background

Respondents were asked to write down their fields of expertise and research interests. Those who had written words inclusive of “*ethno-*” or “*socio-*” as a prefix such as “ethnobotany” or “ethnobiology” were classified as “ethnobiologists.” The word “ethnobiologist” in this study was chosen as a general representative term and is not meant in its strict sense. Terms that did not have these prefixes but were a direct interpretation of social and cultural studies were also put into this classification. The remaining respondents were classified as pertaining to non-ethnobiologists. The purpose of this division was to distinguish those who include social and cultural aspects in their work from those who do not.

#### Personal perspective index (PP index)

We developed a PP index for each study participant as a proxy for their acceptance of non-Western ontological knowledge and beliefs, based on the statements presented in Table 2. Respondents received a score of 1 for every statement they agreed to and a 0 for every statement they disagreed to. The cumulative sum of these scores ranged between 0 (no agreement) to 12 (full agreement). Statements whereby participants had selected “other” as an answer were not taken into account in the analysis.

#### Opinions on plant intelligence

Interdisciplinary opinions of plant intelligence were collected either through direct questions targeted to all participants or only ethnobiologists. We then calculated two types of respondent opinions on plant intelligence. The first was directly obtained at the beginning of the questionnaire as a direct response to the question “*Do you think plants are intelligent*?”; and the second was an aggregate score based on all plant behavioral examples classified into two main groups (“plants are intelligent” or “plants are not intelligent”). Unclear opinions were omitted. The second opinion was derived from a score given to each respondent which represented their tendency to respond to all plant behavioral examples in the same way, either as “plants are intelligent” or “plants are not intelligent.” This score was calculated based on the responses of all Likert-scale questions indicative of plant intelligent behavior. The scores were first normalized due to uneven Likert-scale measurements (some questions had a scale from 1 to 6 while others ranged from 1 to 4). The minimum scale referred to “not an intelligent behavior” and the maximum scale “a very intelligent behavior.” Each response was given equal weight, summed, and then divided by the total maximum score (32). Each respondent was then given a final score ranging from 0 to 1. The median score of 0.68 was used as a threshold to divide the upper and lower half respondents into two groups, those who favored plant intelligence versus those who did not. These two opinions of plant intelligence were then compared to calculate the proportion of subjects whose opinions had remained the same or changed.

### Quantitative analysis

All data were analyzed using nonparametric tests, including Kruskal–Wallis, chi-square (*χ*^2^*)*, Cramer’s *V*, and two-proportion *Z* test. Ordinal data were tested for significance of associations (chi-squared test). Contingency tables, where cells did not meet the chi-square test’s assumptions [62], were corrected with a Yates correction for continuity to reduce the risk of type II errors in order to prevent an overestimation of statistical significance. The standardized residuals of each cell in chi-squared tests were then observed to explore which cells contributed most to the chi-square value. A strength test, the Cramer’s *V* test, displayed how strong the association between the groups was, serving as an equivalent to the Pearson’s correlation test. To understand the relationships between cells, the two-proportion *Z* test was used. *Z* tests were also corrected for multiple testing by using the Yates correction method. Furthermore, Cronbach’s alpha was estimated for groups of Likert-scale questions with different items (all the behavioral examples presented) to test how internally consistent responses would be if participants repeated the questions, i.e., a coefficient of how reliable their responses were [63]. The percent distribution, Likert mean score, and expected mean were calculated for all plant behavioral examples. The mean was then subtracted from the expected mean to observe which direction each response leaned toward, either pro- or anti-plant intelligence views.

### Qualitative analysis

Themes related to respondents’ answers were manually identified in Microsoft Excel due to the limited amount of text (word count) provided in the answers. Co-occurring thematic explanations were counted for each plant behavioral example (Table 4). Thematic categories were then classified and defined by grouping together related themes and repeated words (Appendix Table 6). A particular theme was identified when it was stated by more than three respondents. Statements in which a theme could not be produced or interpreted due to ambiguity, insufficiency in explanatory strength, reduced word count, and/or irrelevance to the subject matter were omitted. In total, twelve co-occurring thematic explanations were compiled based on respondents’ open-ended answers on case studies of plant intelligence.

## Results

### Perspectives of plant intelligence and applications of TK

Seventy percent of respondents reported that they used TK in their field of study less than half of the time, while 30% reported using TK more than half of the time (*n* = 120). Opinions of plant intelligence were significantly associated with the frequency of using TK. A higher number of respondents who reported using TK more often considered plants intelligent (*χ*-squared = 4.091, *df* = 1, *p* value = 0.0431, Cramer’s *V* = 0.21, *n* = 92). Opinions on the value of TK according to plant scientists were significantly associated with professional background, thus separating ethnobiologists (*n* = 30) from non-ethnobiologists (*n* = 111) (*χ*-squared = 8.055, *df* = 1, *p* value = 0.005, Cramer’s *V* = 0.23, n = 141). A larger proportion of respondents (69%) considered that TK has been undervalued by plant scientists, with a larger share of ethnobiologists sharing this opinion. The value of TK was not associated with participant age nor frequency of collaboration outside the biological sciences (thus with the humanities or social sciences). Despite the fact that the majority of respondents did not include TK frequently, 64% agreed that TK could be useful in their field of study and that TK could help close study gaps (53%) (*n* = 143). Respondents who did not consider TK helpful to close study gaps were more likely to be non-ethnobiologists (47%) (*χ*-squared = 22.6, *df* = 1, *p* value < 0.0001, Cramer’s *V* = 0.40, *n* = 143).

### Opinions of plant intelligence

More than half of all respondents (58%) were familiar with the term “plant intelligence.” There was no significant association between opinions of plant intelligence and the following variables: (1) professional background (ethnobiologist or non-ethnobiologist sensu stricto) (*n* = 114); (2) type of profession (professional researcher or graduate student) (*n* = 144); (3) age (*n* = 103); or (4) familiarity with the concept of plant intelligence (*n* = 141). Sixty-nine percent of respondents initially considered plants intelligent, 20% did not, whereas 12% stated that their opinion depended on the definition of plant intelligence (*n* = 123). When comparing respondents’ first and second opinions of plant intelligence (measured, respectively, directly at the beginning and later in the questionnaire as an aggregate scoring based on a set of plant behavioral examples of plant intelligence), only one in five respondents (21%) changed their opinion, whereas most individuals (79%) maintained the same opinion.

The results of the plant behavioral examples had an internal consistency of 0.96 (Cronbach’s alpha) which demonstrated very good reliability of respondents’ answers (Table 1). Sixty-four percent of respondents scored the plant behavioral examples homogeneously (i.e., they responded with the same opinion) (*n* = 114). Ethnobiologists did not differ significantly from non-ethnobiologists in terms of consistency of their answers. Within the latter group, the examples of mere behaviors had more counts of “evolutionary processes” as explanation than the examples of intelligent behavior (Table 4). Furthermore, only two respondents were able to discriminate between true and false plant intelligence behavioral examples. Most behavioral examples of plant intelligence had a higher Likert mean score than the expected mean (2.5 for examples 3–9 and 3.5 for examples 1 and 2), indicating that the direction of the responses leaned slightly toward pro-plant intelligence views (Table 1). However, habituation in *Mimosa pudica* L. was the only exception with a mean Likert score of 3.4. Compared to conditioned learning and other flexible plant behaviors, a habitual response may have seemed less impressive to participants. The two examples of mere behaviors had a lower Likert mean score than expected (2.5), indicating that the direction of responses leaned toward anti-plant intelligence views.

**Table 1:** A list of the plant behavioral examples provided in the questionnaire where respondents were asked to rank on a scale from one to four (or six) whether they considered the presented behavior to be intelligent or not (1 = not intelligent to 4 or 6 = very intelligent)

Plant behavioral examples^a^	% distribution						*n*	Mean^b^
	1	2	3	4	5	6		
(1) Habituation in *Mimosa pudica* L								
Habituation is a decreased response of an individual to a stimulus that is being presented repeatedly (your response to a new sound diminishes as you become accustomed to it; traffic, for instance). Can plants become accustomed to a repeated stimulus? *Mimosa pudica* L. (the sensitive plant) is well known for quickly folding its leaves in response to touch. It is possible that Mimosa exemplars stop folding their leaves after being exposed to frequently repeated stimuli. Gagliano et al. [15] studied habituation in Mimosa by repeatedly dropping potted individuals from a certain height. As they reported, the plants in effect stopped folding after a number of drops. The experiment discarded the alternative hypothesis that the leaves had stopped folding due, for instance, to motor fatigue after the continuous drops, and not to habituation. Intriguingly, this learned response persisted, lasting up to a month [15]	21.1	14.6	10.6	21.1	19.5	13	123	**3.4**
(2) Conditioned learning in *Pisum sativum* L								
In Pavlov’s classical conditioned learning, Pavlov was able to show that a trained dog was able to salivate with just a neutral cue (a bell as the conditioned stimulus) in the absence of its food (the unconditioned stimulus). The same type of conditioned learning was conducted in green pea plants, *Pisum sativum* L. [16]. Blue light was the “food” source, a fan was the neutral cue, and the response was growth toward the stimuli. Results from Gagliano et al. [16] demonstrated that the pea plants were able to associate the wind with where the light was going to be. In the absence of blue light, the plant was still able to associate that the direction of the blowing fan would determine where the light source would be. Not only did the green pea plants have to respond to two external stimuli, but it also had to internally and globally process that those two stimuli were associated. This type of learned behavior can be seen as an expression of an anticipatory behavior that required storing and processing information and globally responding [16]	16.5	9.9	14	22.3	21.5	15.7	121	3.7
(3) Salt avoidance in *Arabidopsis thaliana* (L.) Heynh								
Roots need to make the best overall decision. Apart from growing toward gravity, water, and nutrients, they must sense sources of stress such as salinity. Roots of *Arabidopsis thaliana* (L.) Heynh. Navigate belowground to avoid high saline conditions. The root apparatus possesses a sophisticated sensory and communication system that allows the plant to respond flexibly. Roots must integrate these and many other cues so as to grow one way or another under constantly changing conditions [23]	23.3	21.6	35.3	19.8			116	2.5
(4) Host preference in parasitic plants								
The parasite dodder (*Cuscuta pentagona* Engelm.) obtains its nutrients from the shoots of the host plant it climbs onto. By sensing the airborne volatile compounds that potential hosts emit, they are able to choose the more nutritious ones. In the vicinity of a tomato and a wheat plant, the dodder can tell them apart and grow toward the more nutritious tomato exemplar [25]	22.4	19.8	37.9	19.8			116	2.5
(5) Mycoheterotrophic parasitic plant (mere behavior)								
Mycoheterotrophic plants cannot do photosynthesis. They obtain all their water and nutrients from the fungi they parasite providing nothing in return. Many of them mimic fungi varieties other than their host, remaining belowground and becoming visible only when they flower. Their metabolic cost in doing so is minimal [54]	32.2	20	33.9	13.9			115	**2.3**
(6) Secondary metabolite manipulations of nectar in plant–ant relationships								
Many plants secrete extrafloral nectaries as food for ants in return for their protection. Plants need to attract the right kind of ant partner and actively maintain their quality protection. The nectar is custom-modified to specific ants. Too little nectar can discourage ants away while providing too much nectar can lower the quality of protection. These plants have to sense the presence of different ants, monitor and modify their activity accordingly in order to get the best benefits [19]	21.7	18.3	37.4	22.6			115	2.6
(7) Resource exploitation by roots								
In one study, roots belonging to the same pea plant (*Pisum sativum* L.) were exposed to two patches of nutrient: one patch with more amount of nutrients but whose concentration remained constant, and a second patch with less amount of nutrients, but whose concentration would increase throughout the experiment. The pea plants grew more roots to the patch that currently had less amount of nutrients, but would have more in the future due to the steady increase [26]	25.2	16.5	41.7	16.5			115	2.5
(8) Plant defense turning herbivores into cannibals (mere behavior)								
Some tomato plants (*Solanum lycopersicum* L.) are able to defend themselves against herbivory by releasing certain chemicals (methyl jasmonate). Researchers observed that spraying the tomato leaves with methyl jasmonate promoted cannibalism: munching caterpillars would prefer to change diet and eat other fellow caterpillars instead [55]	33.3	16.7	28	21.9			114	**2.4**
(9) Numerical sensitivity and short-term memory: Venus fly trap, *Dionaea muscipula* J.Ellis								
The Venus flytrap, *Dionaea muscipula* J.Ellis, snaps shut when it gets mechanically stimulated twice. After the first stimulation, the second needs to occur within 20 s of the first to snap shut. If it does not, then it resets. While counting to two may seem less impressive, counting to five is more difficult to do. Before digestive enzymes are secreted, the Venus fly trap needs to keep counting the numbers of mechanical stimulation until it reaches five. It is the plant’s extra security mechanism. *Dionaea* spp. are able to count, store information as a form of short-term memory, and repeat the process [14]	27.2	13.1	40	23.4			114	2.7

^a^Response scale 1 = not intelligent to 4 (or 6) = very intelligent

^b^Mean Likert scores higher or lower than the expected mean (3.5 for examples 1 and 2 and 2.5 for the rest) indicate pro-plant intelligence views and anti-plant intelligence views, respectively

Numbers in bold indicate mean Likert scores lower than the expected mean (i.e., anti-plant intelligence views)

### Respondents’ personal perspectives (PP index)

Table 2 reports respondent agreement or disagreement with the individual statements as well as an aggregate opinion on plant intelligence by combining all plant behavioral examples that were indicative of plant intelligence. Statements 3 and 5 had the largest Cramer's *V* value (0.48 and 0.47, respectively), indicating that concepts related to plant communication and plant response to sound have a relatively stronger association with views on plant intelligence. The remaining Cramer’s *V* values had a relatively poor strength of association, suggesting that concepts less specific to human–plant interactions are poorly associated with individual opinions of plant intelligence.

**Table 2:** The number of respondents who agreed or disagreed with the statements and their aggregate opinion on plant intelligence measured by combining all plant behavioral examples indicative of plant intelligence. All statistically significant results had statistically significant pairwise comparisons (two proportion *Z* test)

Perspectives	Combined opinion of plant intelligence			Chi-square value	Cramer's *V*	*p* value	*n*	References
		Intelligent	Not intelligent					
1. A plant or tree has spoken to me before	Agree	11	2	5.597	0.23	0.018	108	[46]
	Disagree	43	52					
2. I believe that hallucinogenic drugs reveal inner truths of an individual and other truths of the world. These “hallucinations” are not irrelevant	Agree	25	9	7.143	0.27	0.008	94	[56]
	Disagree	28	37					
3. I believe singing or talking to plants doesn't have any effect on the plant	Agree	12	34	22.809	0.48	< 0.001	100	[57, 58]
	Disagree	41	13					
4. I don't believe in the significance of dreams; they do not hold any factual truth about the world	Agree	15	27	8.719	0.29	0.003	103	[42, 46]
	Disagree	41	20					
5. I believe that the Flora kingdom can communicate with us, either through dreams, visionary illustration and/or other means of communication	Agree	35	7	23.343	0.47	< 0.001	105	[59, 60]
	Disagree	21	42					
6. I believe that plants are teachers	Agree	40	23	4.537	0.21	0.033	103	[46, 60]
	Disagree	16	24					
7. I would consider myself spiritual. By spiritual, it does not necessarily conform to a labeled religion	Agree	36	16	7.143	0.27	0.005	107	[49, 51]
	Disagree	22	33					
8. I don't believe in the existence of a spiritual, unseen dimension	Agree	31	17	5.725	0.24	0.017	100	[49, 51]
	Disagree	21	33					
9. I practice mindful meditation when I can. I believe its practices allow me to connect with my higher self	Agree	34	44	11.478	0.32	0.003	115	[49, 51]
	Disagree	25	6					
10. I incorporate Eastern philosophy into my way of living such as Taoism, Buddhism and Hinduism	Agree	16	7	5.597	0.16	0.033	111	^–^
	Disagree	41	47					
11. I am a person who listens and follows what my heart says relatively more than my mind	Agree	21	13	2.465	–	0.29	99	[61]
	Disagree	27	34					
12. I feel strongly and personally connected to nature	Agree	57	45	3.276	^–^	0.19	117	[5]
	Disagree	3	7					

Ethnobiologists (*n* = 15) had a significantly higher PP index (reflecting acceptance of non-Western ontological knowledge and beliefs) than non-ethnobiologists (Kruskal–Wallis chi-squared = 12.8, *df* = 1, *p* value = 0.0004, *n* = 66). A higher PP index was also significantly related to those who have a higher frequency of collaboration outside the biological sciences (Kruskal–Wallis chi-squared = 12.238, *df* = 1, *p* value = 0.0004, *n* = 56) and within the humanities (Kruskal–Wallis chi-squared = 4.368, *df* = 1, *p* value = 0.037, *n* = 53).

### Responses on analogous behavioral examples in plants

Seventy-six percent of respondents scored the animal behavioral examples of camouflaging in a cephalopod and climbing behavior of macaques as intelligent. Others had either mixed opinions (20%) (i.e., intelligent in one example and not intelligent in the other example), or did not think these behaviors were intelligent (4%). More respondents considered the *Corydalis hemidicentra* camouflaging behavior and *Syngonium* spp. foraging behavior as “not intelligent” (Appendix Fig. 2). Approximately 32% of respondents shifted their opinion from “this is an intelligent animal behavior” to “this is not an intelligent plant behavior.” Internal consistency (Cronbach’s alpha) of answers was 0.8.

The mean Likert scores for the camouflaging and foraging behavior in animals were 4.2 and 4.7, respectively, indicating pro-intelligence views. Furthermore, the corresponding plant camouflaging and foraging behavior had a mean Likert score of 2.5 and 2.6, respectively, indicating a marginal (or neutral) view of intelligence (Table 3).

**Table 3:** A list of behavioral examples provided in the questionnaire where respondents were asked to rank on a scale from one to four (or six) whether they considered the presented behavior to be intelligent or not (1 = not intelligent to 4 or 6 = very intelligent)

Behavioral Examples^a^	% distribution						*n*	Mean^b^
	1	2	3	4	5	6		
*Animal behavior*								
*Camouflaging*: Cephalopod species (squids, octopus and cuttlefish) are known to be very intelligent invertebrates. The octopus have evolved an effective and impressive camouflaging ability that allows them to manipulate and exploit their surroundings to hide from predators and hide from prey. This intelligent behavior requires the ability to acquire external information, process complex information, and flexibly adapt in order to successfully achieve its goal [64]	6	3.4	11.2	21.6	31.9	25.9	116	4.2
*Foraging*: The omnivorous stump-tailed macaques, *Macaca arctoides,* spends half of its day foraging and feeding. *M. arctoides* usually travel on the ground, but do climb trees either to forage for food resources or to go to sleep. They travel to areas where food is abundant, by remembering the exact locations and by following the changes in seasonal patterns of the vegetation. They can travel from tree to tree when they forage, avoid other territories belonging to other groups, and descend to continue to forage in other areas when food resources have been eaten up. Many climbing species, much like *M. arctoides*, forage for food in an adaptable, meaningful and flexible manner. They know where resources are, avoid competition, and anticipate where food resources will be. They respond to these changes to ultimately maximize their fitness [65]	0.9	2.5	2.5	17	38.1	39	118	4.7
*Plant behavior*								
*Camouflaging*: Some plant species (*Corydalis hemidicentra* Hand.-Mazz) can camouflage like animals with the same known behaviors: (1) Background matching—blending with the colors of shapes of the habitat where they live; (2) Disruptive coloration—markings that create the appearance of false edges and boundaries, making it harder to see the true outline; (3) Masquerade—looking like something else; usually something a predator might ignore, such a stone or twig. Examples include living stones, some cacti, passion vines and mistletoes; and (4) Decoration—accumulating material from the environment. For example, some coastal and dune plants get covered by sand because of their sticky glandular trichomes, making them less conspicuous. This intelligent behavior requires the ability to acquire external information, process complex information, and flexibly adapt in order to successfully achieve its goal [66]	18.9	30.6	30.6	19.8			111	2.5
*Foraging*: Climbing plants, such as tropical species from the Araceae family, *Syngonium* spp., forages for light by reaching the top of its host tree and descends to the ground while searching for another host tree. Its morphological features also progressively changes as it climbs, growing thicker filiform stems (tendrils) with larger and thicker leaves at the top. The tendril explores to find other tree hosts by extending and descending downwards, having thinner tendrils and smaller leaves. *Syngonium* spp. can therefore switch between mobile and sessile behavior. It has the capacity to decide when to climb and when to descend, by flexibly changing its growth direction given exogenous changes. This light-foraging behavior is adaptable and meaningful [67]	13.5	29.7	38.7	18			111	2.6

^a^Response scale 1 = not intelligent to 4 (or 6) = very intelligent

^b^Mean Likert scores higher or lower than the expected mean (3.5 for examples 1 and 2 and 2.5 for the rest) indicate pro-plant intelligence views and anti-plant intelligence views, respectively

### Qualitative results

#### References to “plants being intelligent” by local people

One of the questions ethnobiologists were asked was “*Have local people you worked with ever mentioned in their own way that plants are intelligent? If so, how*?”. Fourteen respondents shared their field experiences. The main concepts ethnobiologists had recorded from local community members were: (1) plants have emotions, (2) plants can communicate with other plants and animals, (3) plants are conscious beings, (4) plants are considered kin, (5) plants are divine beings and have spirit(s), (6) plants need respect and require offerings, and (7) people can communicate with plants to learn from them and collect natural resources.

#### Plant subjective awareness

Subjective awareness was defined as “*involving the ability to be aware, aware of one’s own body parts and existence*.” To the question “*Do you think plants can be subjectively aware?*,” 42 participants responded with a dichotomous “yes” (*n* = 17) or “no” (*n* = 25). In addition, 77 responses pertained to seven themes (number of participants given in brackets): It depends on the definition (11); uncertain (28); yes, in a non-anthropocentric way (8); yes, as an intrinsic ability (11); no, there are no neuronal networks or thought patterns involved (10); open to the possibility (4); this cannot be proven or disproven (5).

#### Plant behavioral examples

Several respondents explained why they considered the examples intelligent behavior, despite the open question asking the contrary (i.e., why is this example not considered an intelligent behavior?). Explanations can be summarized under the following themes: (1) brains are not necessary for intelligence, (2) adaptation to the environment to maximize fitness is intelligent, (3) intentional behavior for survival is intelligent, (4) successful strategies and adaptable behavior require intelligence, and lastly (5) it is an *intrinsic* trait of a plant. They agreed that learned behavior, having memory, and the ability to make associations are a form of intelligence. In contrast, other respondents stated that basic learning, having memory, and the ability to make associations are insufficient to be qualified as a form of intelligence. Thus, explanations were diverse and showed divergent views. Table 4 gives an overview of all thematic explanations for each of the behavioral examples. Frequently, explanations included reasons that implied mechanical processes exhibited without any “intention” or “awareness” from a plant. Another common theme of explanations was that the adaptive behaviors made by plants are fixed and controlled by genes, an evolutionary power that is not related to individual decision-making.

**Table 4:** A summary of the main co-occurring thematic explanations of individual responses for each of the plant behavioral examples asked in the questionnaire

Co-occuring thematic explanations	Habituation in *Mimosa pudica* L	Conditioned learning in *Pisum sativum* L	Salt avoidance in *Arabidopsis thaliana* (L.) Heynh	Host preference in *Cuscuta pentagona* Engelm	Nectar in plant–ant relationships	Resource exploitation by roots of *Pisum sativum* L	Counting and memory in the Venus fly trap	Mycoheterotrophic plants	Tomato plant turns herbivore into cannibals
Uncertain	–	5	2	3	1	1	3	2	2
Response to external stimuli	4	4	7	2	1	2	1	–	–
Depends on definition	4	3	2	2	1	1	–	1	1
Physiological response	6	3	3	2	–	1	2	–	1
Automated response	4	1	8	9	7	3	5	5	3
Evolutionary process	1	–	2	6	17	3	8	26	17
No conscious thought involved	3	1	8	6	5	3	6	6	3
Chemical response	–	–	1	5	–	1	–	–	5
Threshold response	–	–	–	–	–	–	5	1	–
Tropism	–	–	4	3	–	2	–	–	–
Expresses concern about the study	–	4	6	–	2	4	–	–	–

	Case-specific themes								
	Saving energy						Not considered counting	Mimicry/parasitism is not intelligent	Response not directed by plant
	3						5	4	7
Total number of respondents	53	45	49	47	42	42	43	57	52

The numbers represent the occurrence of one theme being mentioned per behavioral example. Case-specific explanations were themes unique to a behavioral example. A theme was only produced if it was mentioned more than three times in any of the behavioral examples. For the definition board where all themes were explained, see appendix Table 6. The last two behavioral examples on the right were behaviors not intended as indicators of plant intelligence but, instead, of mere adaptation

A small number of participants expressed confusion about the definition of intelligence or stated that intelligence is a semantic issue. Some people showed expressions of conflict and uncertainty by explicitly stating that they could not provide an opinionated response while some also questioned or reserved the meaning of intelligence exclusively for humans or humans and other animals. In addition, some were uncertain what constitutes intelligence (and the levels of intelligence) and how it can be theoretically applied to plants.

The explanations summarized in Table 4 demonstrated that the thought patterns of plant scientists can be divided into three main groups. One subgroup paid more attention to the mechanistic properties as a determinant of intelligent behavior. A second subgroup was concerned less with the mechanical processes, but interpreted these behaviors as controlled by genes which, due to evolutionary processes, determined beneficial adaptive behaviors. A third subgroup rather relied on what the behaviors represented as a whole, that is whether the observed behaviors conceptually indicated intelligence or not.

## Discussion

**Fig. 1:**
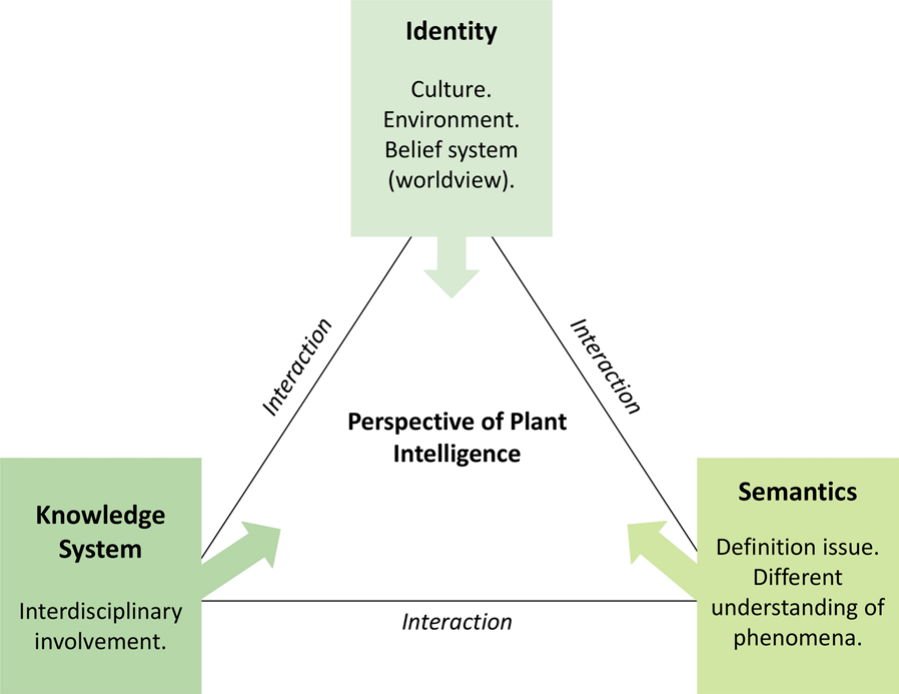
A conceptual framework on the components that can influence a researcher’s perspective of plant intelligence

The subtle but crucial variations that differentiate mere plant behaviors from intelligent behavior were not noticed by most respondents. Such mere behaviors are responses that are fixed, i.e., genetically determined, and incapable of long-term change such as the example of mycoheterotrophic plants in Table 1. One possible explanation for this could be that a considerable degree of familiarity with the concept of plant intelligence is required to detect these subtleties. We tried to make up for this by introducing the concept of plant intelligence briefly prior to the start of the questionnaire. However, it likely requires training and experience to interpret and compare different types of plant behaviors. Regardless, our results show that there exist significant gaps in knowledge and communication about plant intelligence. These gaps are caused by uncertainties about what entails intelligent plant behavior and how it can be distinguished from other behaviors. Our results highlight the complexity and diversity of opinions on plant intelligence and show that an individual view of plant intelligence depends on several factors. As a result, based on our findings supplemented with the literature on factors contributing to the lack of consensus on plant intelligence [33], we developed a conceptual framework (Fig. 1) consisting of three key components and their factors that influence a scientist’s perspectives. This conceptual framework aims to explain why variations in perspectives of plant intelligence occur and highlights the gaps in knowledge and communication observed in our study.

### Identity

Central to the conceptual framework are factors that contribute to the formation of a person’s identity, namely culture, environment, and beliefs. Culture plays a key role in valuation systems and decision-making processes [68, 69]. Interactions between humans and plants have significantly governed cultures and the surrounding landscape (e.g., [70]). Cultures have been conceived and continuously shaped by the various ecosystem services of plants (and nature as a whole). Culture contributes to the perceptual lens by which natural phenomena (in this case, plant behaviors) are observed and interpreted. Furthermore, the human–nature relationship is reflected through relational processes, for example, one’s sense of place, value systems, and connections to nature [71, 72], and lived experiences between people and the environment [4]. Culture and environment are therefore tightly interlinked and mutually shape each other [73]. Finally, an individual’s belief system is inevitably tightly interlinked with culture and the environment. A belief system is the philosophical foundation of how one decides to act within and relate to the environment [72]. As observed in this study, a belief system that either associates or disassociates with belief in the agency of plants, either implicitly or explicitly, reflects a scientist’s perspectives of plant intelligence, independently from existing scientific evidence. Our findings conform to claims made by other authors (e.g., [74]) that disagreements and non-recognition of plants’ behavioral ability had less to do with scientific evidence but more with personal belief systems, regardless of the evidence provided. However, we acknowledge that plant intelligence is still a relatively new concept with minimal supporting experimental studies—e.g., independent replication of Gagliano and coworkers’ pea plant associative learning experiments is still lacking [75]; although see [76, 77]—and, evidently, will result in disagreements and miscommunication in the interpretations of various plant responses.

Both the environment and belief systems are embedded within a broader cultural dimension and constitute one’s worldview [78]. Here, a worldview is described as an intuitive view, a set of values, and belief system concerning one’s conception of life [78]. These three factors (culture, environment, and belief system) take part in the formation of a plant scientist’s professional identity, the knowledge system they hold onto, and interpretations of ecological phenomena. Therefore, interpretations of plant behaviors evidently reflect a scientist’s subjective opinions about plant intelligence, either implicitly or explicitly.

### Knowledge systems

Respondents came from a variety of professional backgrounds, most of which do not require the involvement of other forms of knowledge (such as TK) in their work. However, involvement with TK systems and multidisciplinary collaborations may provide a complimentary and inspirational lens for scientific investigations and is even more essential when decision-making in resource management is involved [47, 48, 79–82]. In plant-related studies, it expands the ways scientists understand different plant behaviors more holistically, taking into consideration both mechanistic properties and the overall representation of ecological phenomena of an observed plant behavior. Our findings showed that plant scientists who more frequently included TK in their discipline agreed more with the idea of plant intelligence.

Interestingly, one study showed that social scientists have more of an organismic worldview (more holistic, interactive with, and integrated into social environments), while natural scientists have a more mechanistic worldview (more objective, reductionistic, and separate from social environments) [83]. Authors in [84] discussed how social and biophysical sciences have different levels of awareness about the relationship of cultural worldviews shaping scientific theory and practices. Our study provides a similar finding where two groups of plant scientists from different backgrounds (either ethnobiologists or non-ethnobiologists) also differed in their ontological views of nature and plants, especially with specific ontologies that are associated with traditional knowledge of plants. Plant scientists who included sociological aspects in their work and collaborated more frequently outside their disciplinary field tended to associate more positively with non-Western knowledge systems than sensu* stricto* plant scientists. These associations therefore take part in influencing a plant scientist’s perceptions of plant intelligence.

### Semantics

The language and meaning that is used to describe plant behaviors affects the way information about plant intelligence is being communicated and understood. Consequently, the majority of arguments and disagreements can largely be attributed to semantic issues and not the phenomena of plant behaviors. Some plant scientists may take the terminologies describing plant behavior literally when words are used as metaphors, such as the phrase “plant neurobiology” [85, 86] or metaphorically when words were meant to be taken literally, such as the phrase “plant intelligence” [87].

There are multiple definitions for “intelligence” without a current consensus [88]. This poses a problem where disputes will be mostly about the meaning of plant intelligence and determining what definition and theoretical framework of intelligence can be best applied to plants. Another issue is whether there should be a unified framework for intelligence, or whether different frameworks for intelligence should be allowed to exist [6, 89]. Different theoretical frameworks of intelligence will use their own terminologies to discuss their theories, approaches, and discoveries. Definitions (and theoretical frameworks) are also not set in stone and are subject to ongoing revision. Even when definition issues have been resolved and all parties have the same understanding of the meaning of intelligence and settle on the same theoretical framework, differences in understanding the same phenomena will still exist. This was also observed in our study, given that the definition of intelligence was provided prior to the questionnaire. According to respondents, the plant behaviors provided in Table 1, such as complex decision-making, anticipation, counting, and adaptability, were interpreted differently with explanations in favor or against intelligent behavior.

Problems with semantics and personal subjectivity are also common in other biological sciences, exemplified by the definition and diversity of perspectives about “invasions” in biological studies [89]. Therefore, open discussions are needed so that different subjective viewpoints can be shared, an end goal known as intersubjectivity. For example, the author in [90] discussed how achieving intersubjectivity can act as an intermediary by actively coordinating participants who disagree with each other to express their subjective opinions. As a result, this can allow communicated viewpoints to be less subjective.

### Future implications

The scientific paradigm of plant intelligence can benefit from the integration of ethno- and other social sciences, because of their relationship with multiple worldviews. The concept of plant intelligence has been mentioned in various socially oriented disciplines, e.g., philosophy [7, 91, 92], ethnobotany [93], and anthropology [94, 95]. Meanwhile, to our knowledge, only a few literature references in the biological sciences incorporate the ontological features of plant intelligence (e.g., [96, 97]). The results of this study make the case for facilitating an interdisciplinary dialogue within various plant-related disciplines among those that argue for and against plant intelligence, so that these scientists may take away multiple perspectives and adopt them during their scientific practice [47]. Researchers will be able to express their personal viewpoints and explain their unique interpretations of plant behaviors. Such dialogue allows for self-expression and respectful consideration of various perspectives that stretch beyond an individual’s boundary of knowledge and acceptance (i.e., paradigm) and toward a more holistic view of plants. It also offers new opportunities for creative, complementary approaches and collaborations to be conceived. There exist various methodologies to observe whether (and how) people can change their attitudes (and therefore beliefs) through strategic ways of communication [90]. Furthermore, a future systematic in-depth compilation of candidate plant intelligent behaviors could become a reliable basis for future scientific inquiry.

### Study limitations

The strength of our results depends on the quality of responses and attentiveness of respondents. The time it took participants to finish the questionnaire ranged widely, from 20 min to 2 h. Repetitive responses may have partially reflected respondents’ waning interest, or point to structural issues with the questionnaire. The fourth research question could not be answered conclusively through our questionnaire. There is room for further development of our psychometric scale, which was a pilot design for a new study topic. Psychological metrics worth considering in future studies are those that address in detail (1) multiple relationships between people and plants; (2) intrinsic and instrumental valuations of other organisms; (3) the degree of understanding ecological interconnectedness; and (4) the level of trust and credibility in both scientific and traditional knowledge, which would potentially provide further insight into what types of ontologies shape researchers’ opinions of plant intelligence. Finally, our study was unable to test perspectives of plant intelligence as a general function (or model) of interactions between the three key components (and factors that contributed to each of them) of our conceptual framework (Fig. 1). We were limited to testing two variables (or groups) at each time independently. Future studies can elaborate on this. Regarding definitions of terms, our questionnaire did not clearly define what “using” TK systems in one’s field of study meant. As a result, we were unable to ascertain how respondents “used” TK in the way the study intended, as a respectful integration of different forms of knowledge.

## Conclusions

This study demonstrates that the science, language, and subjectivity of participants all take part in forming and formulating their opinion of plant intelligence; therefore, these should not be considered or treated independently. Through the conceptual framework presented in this study, we hope to demonstrate that the understanding of plant intelligence extends beyond its dependence on scientific definition and knowledge. It is challenging to separate personal worldviews and thus subjectivity from conceptualizations and interpretations of plant behaviors in an academic environment. In addition, our results show some openness and support for intelligent behavior in plants while also, in general, showing a dependence on the conceptual framework and definition of intelligence. In conclusion, effective communication and an interdisciplinary dialogue among a wide range of interested scientists are actions that can help to close the divide and promote mutual understanding and consensus on plant intelligent behavior. Increased openness for and respectful considerations of TK can be a positive influence for plant scientists when interpreting plant behaviors in favor of intelligence. With growing respect and acknowledgement of diverse perspectives of plant intelligence, new research questions and approaches can be opened for future scientific investigation.

## Figures and Tables

**Fig. 2:**
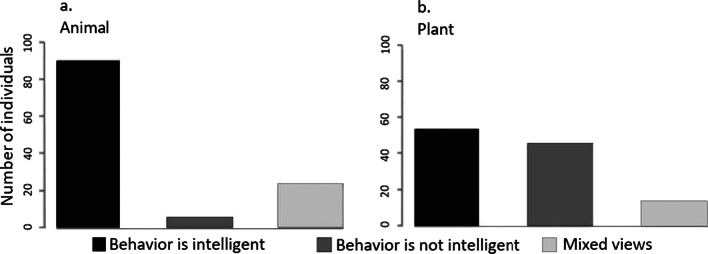
The number of participants who responded to the **a** two animal behavioral examples combined (*n* = 118) and their **b** corresponding analogous plant behavioral examples (*n* = 111). The animal behavioral examples were of cephalopod camouflaging and Macaques foraging behavior, while their corresponding analogous behaviors in plants were of *Corydalis hemidicentra* and *Syngonium* spp., respectively

## Data Availability

The dataset used and/or analyzed during the current study is available from the corresponding author on reasonable request.

## Appendix

See Fig. 2 and Tables 5 and 6.

**Table 5:** List of keywords entered in online search tools to find e-mails of target participants

Google Search	Google Scholar	Researchgate.com	Academia.edu	Journal of Ethnobiology and Ethnomedicine
Department of plant ecology	Tropical plant ecology	Plant ecology	Plant ethnography	Plant traditional knowledge
Department of botany	Plant physiology	Botany	Ethnomedicine	Magical plants
Tropical research insititute	Indigenous knowledge plants	Ethnobotany	Plant ethnobiology	Medicinal plants
Ethnobotany	Shamanic healing	Plant science	Medicinal plant	Sacred forests
Plant science research group	Medicinal plant knowledge	Plant physiology	Forest dynamics	Plant spiritual belief
Forest ecology group	Anthropology plants	Plant behavior	Plant ecology	
Tropical plant ecology	Ethnology plants	Plant interaction		
Department of biology	Ethnobotany ritual plants			
Ecosystem ecology research groups	Ethnobotany medicinal plants			
Plant ecology research group	Ethnobotany			
Center for enthnobotany	Indigenous cosmology			
Botanical groups	Plant traditional knowledge			
Ethnobotany people university	Plant signaling behavior			
Ethnobotany people instituion				
Ethnobotany abstract books				
Plant science abstract books

**Table 6:** A definition board of how a theme was produced and its interpretation

	Inclusive of words and/or body of text	Interpretation
*Co-occuring themes definition Board*		
Uncertain	“uncertain,” “not sure,” or any body of text that expressed uncertainty	The presented behavior was unclear to provide an opinionated response
Response to external stimuli	“reaction to the environment,” “response to external stimuli,” or any body of text that was interpreted as such	The presented behavior was due to a response to an external stimuli
Depends on definition	“depends on definition,” “definition,” “depends on what is intelligence,” and/or a body of text that was interpreted as, or shown as an expressions of such	A raised concern and/or expression that the presented behavior was dependent on how one defines intelligence and/or plant intelligence
Physiological response	“physiological,” “physiological response,” or interpreted as such	The presented behavior was due to a (hardwired) physiological response
Automated response	“automated response.” “motor reflex,” “reflex,” “hard-wired response,” “instinct,” “direct response,” or interpreted as such	The presented behavior was due to an automated, hardwired response
Evolutionary process	“evolution,” “evolutionary process,” “natural selection,” “selection,” “Darwin,” “evolved,” “genetic evolution,” or interpreted as such	The presented behavior was (more likely) due to an evolutionary process
No conscious thought involved	“no thought involved,” “no conscious thought,” “no active decision/choice,” “no awareness,” “no mental choice involved,” or interpreted as such	The presented behavior did not involve any conscious action(s) made by the plant
Chemical response	“chemical response,” “biochemical response,” “chemical reaction”	The presented behavior was due to a chemical response
Threshold response	“threshold response,” “accumulation of action potential”	The presented behavior was due to a (chemical-based) threshold response
Tropism	suffix “-tropism,” “tropism,” “taxis”	The presented behavior was more or less due to tropism
Not considered counting	“this is not counting,” “not considered counting”	The presented behavior was not considered as a counting strategy/mechanism exhibited by a plant
Mimicry/ parasitism is not intelligent	“mimicry is not intelligent,” “camouflaging is not intelligent”	The presented mimicry is not considered intelligent
Response not directed by plant	“response not directed by plant,” or interpreted as such	The presented behavior was a response not actively directed by plant

## Abbreviations

TK: Traditional knowledge
TKS: Traditional knowledge systems
PP index: Personal perspective index
